# Supplementary material for: Spatial Variation in Stream-Water Bacterial Communities Across Sites Differing in Wild Chinese Giant Salamander Detection Frequency
Source: Biology (Basel). 2026 Sep 18;15(18):1647. doi: 10.3390/biology15181647 (PMC13604818; doi:10.3390/biology15181647)
Supplement: Supplementary file 1 [file biology-15-01647-s001.zip › Data Related to “Spatial Variation in Stream-Water Bacterial Communities across Sites Differing in Wild Chinese Giant Salamander Detection Frequency”/Microbial Data/result/04.AlphaDiversity/sample/visual_rarefaction/simpson/simpson.sample.pdf]

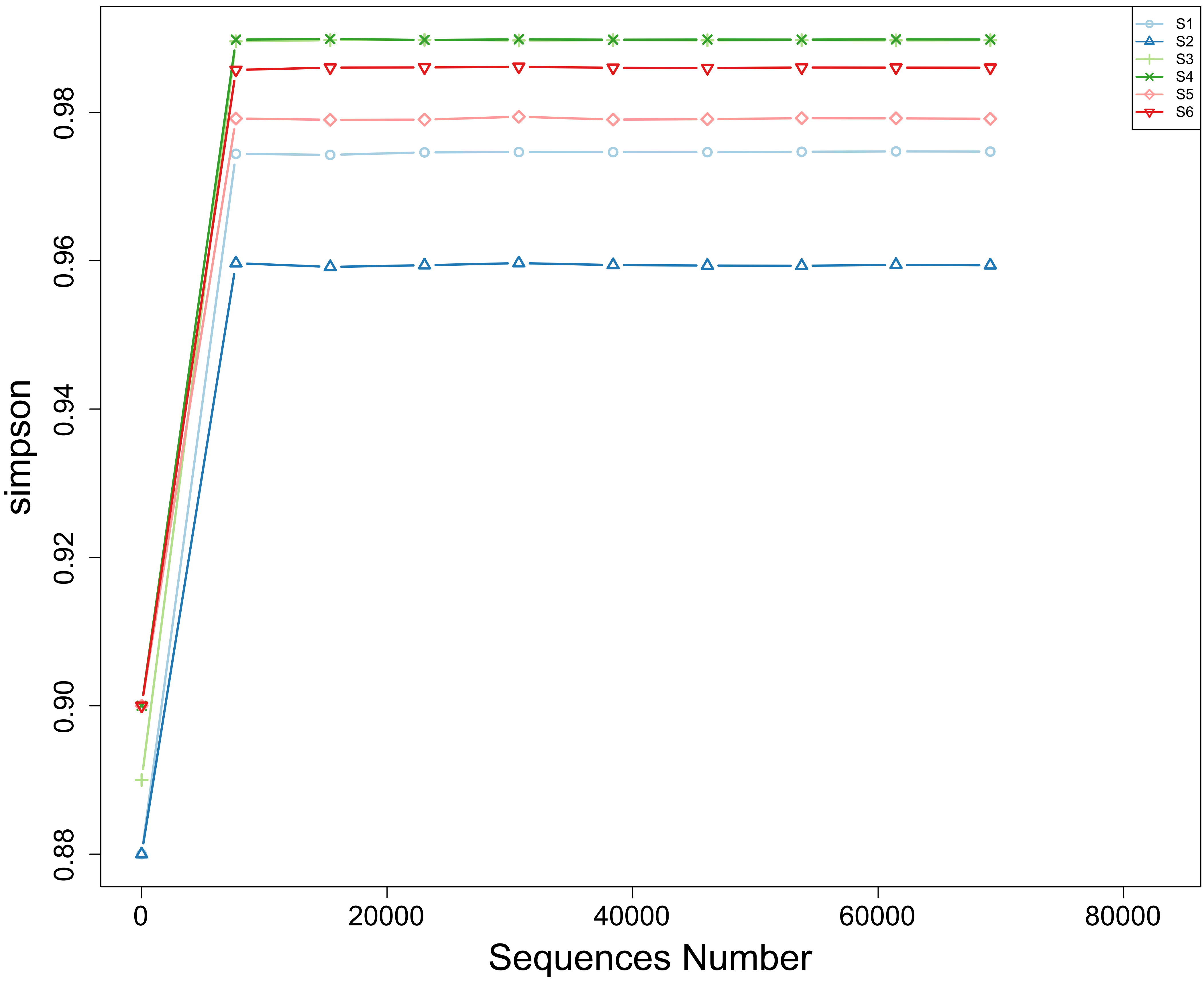

S1
S2
S3
S4
S5
S6
simpson
0.88
0.90
0.92
0.94
0.96
0.98
0
20000
40000
60000
80000
Sequences Number
